# On the Medicinal Effects of the Persesquinitrate of Iron

**Published:** 1848-08

**Authors:** 


*On the Medicinal Effects of the Persesquinitrate of Iron.*—Dr. Graves states* that he has lately used the persesquinitrate of iron with very considerable success. He proceeds to say, that "you will be consulted by females of a delicate and weakly habit, who frequently exhibit symptoms of nervous derangement, such as palpitations, sleeplessness, and headache, who are easily excited or alarmed, have a tendency to emaciation and paleness, and have little or no appetite. Combined with these general symptoms you find that they have been laboring under diarrhœa for weeks, and even for months, and that this with other causes of debility has rendered their condition extremely uncomfortable. You will also be informed by the patient, that she has tried many remedies without benefit, and that she is extremely anxious to have something done to give relief; and hence it is a matter of importance to be acquainted with any remedy which may be likely to prove serviceable. This form of dia.rhœa is of an unmanageable character, and very seldom amenable to the ordinary modes of treatment. The common astringent remedies totally fail; chalk-mixture, kino, rhatany root, and catechu are useless, and in such cases it has been observed, that opium is generally injurious. If you prescribe opium it certainly checks the disease for a time; but this temporary relief is accompanied by debility, malaise, restlessness, and many uneasy symptoms, and the diarrhœa soon returns and is as bad as ever. The medicine which I have found most effectual in such cases is the persesquinitrate of iron. With it I have succeeded in curing many cases which had been exceedingly obstinate, and of very considerable duration, the disease having, in one instance, resisted all the efforts of medical skill for seven months, and in the other for two years. Seven or eight drops of the liq. ferri persesquinitratis, increased gradually to twelve or fifteen in the course of the day, was the quantity prescribed in both cases. In the course of four days a slight diminution of the diarrhœa was perceived, in a fortnight the patient felt much better, and in a month or five weeks it had disappeared altogether. This took place without being followed by any bad effects; there was no swelling of the stomach, no tympanitis, no tormina, no restlessness or nervous derangement; the patients recovered their health and strength, and the cure was at once, safe and permanent."

Cases have occurred to myself where the disease has existed many years, yet were perfectly cured in a surprisingly short time from the use of the persesquinitrate of iron. A great deal of ill health frequently proceeds from chronic diarrhœa. Epilepsy and hysteria, for instance, often exist together, with a long continued relaxed state of the bowels, and do not yield till the remedy is obtained for the diarrhœa. I have seen these and other diseases cured chiefly in consequence of the persesquinitrate of iron checking the accompanying diarrhœa.

Dr. Neligan says, that "the persesquinitrate of iron is an admirable astringent, possessing also tonic properties. It will be found particularly useful in chronic cases of mucus diarrhœa, when there is much emaciation and loss of appetite. In such cases, I have derived much benefit from its employment after many other remedies have failed."†

---

*System of Clinical Medicine, 1843.

†Medicines; their Uses and Modes of Administration, 1847.

In some cases where the ordinary doses (fifteen to thirty drops) thrice a day aggravated the diarrhœa, I have succeeded by giving for some time smaller doses, such as five drops twice or thrice a day, gradually increasing the dose as the patient was able to bear it. In a few cases of the same kind, persesquinitrate has been given in a very large doses by enema. I have already said, that it is generally inapplicable in phthisis. In my paper in the *Edinburgh Medical Journal*, there is a striking instance of a lady in the last stage of phthisis having diarrhœa stopped by enemata containing this medicine. Intestinal ulcerations, if they existed, were probably beyond the reach of the enemata. Mr. Tindal, of Glasgow, informs me of a case of phthisis, where after the failure of many remedies, the diarrhœa uniformly ceased on the patient using the persesquinitrate of iron. These however, are exceptions, and the rule is as I have stated.

I have seldom employed the persesquinitrate of iron in recent attacks of diarrhœa, chiefly because these cases are readily controlled by other medicines. When used in such cases, it succeeded best in large doses, such as half an ounce or an ounce given by enema. With the exception of a few cases of children, its administration in cholera in my hands was a failure. As one cause of this possibly arose from its speedy rejection by vomiting or purging, it has occurred to me, since the departure of cholera from this country, that benefit might perhaps be derived from injecting it, largely diluted with water, into the veins, in the same manner as saline injections.

I have lately given the persesquinitrate of iron internally, with much benefit, in several cases of urticaria. A lady, twenty years of age, who had been afflicted with it since her childhood, and had tried various remedies, without success, was cured by the ordinary doses in less than a month. She is now able to take any article of food, though before using the persesquinitrate she required to be very cautious, and even with the utmost care was seldom a day free from the eruption, and accompanying uneasiness at the stomach.

A lady twenty-one years of age, had an itchy eruption on the face and hands for at least twenty years. When I saw her these parts were swollen, tender looking, and readily exuded a watery lymph. The disease was generally aggravated after dinner, more especially if the dinner was tempting; at this period of the day she retired to her own room, conscious that her appearance was repulsive even to her nearest relatives. It is easy to conceive the unhappiness to which this must have given rise; and though medical men, both in Glasgow and Edinburgh, were consulted at different periods, no plan of treatment was of any avail. After commencing the persesquinitrate of iron, an amendment was soon perceptible; the swelling of the face and hands began to abate, the skin became less tender and less affected by meals, and in the course of some months, a cure was gradually accomplished. General bodily uneasiness was of daily occurrence, and agravated very considerably the depressing effect of the complaint; no symptom was more immediately under the contol of the persesquinitrate, which always made her in a short time more comfortable. I believe that the cure of cutaneous diseases by this medicine is chiefly attributable to its efficacy in improving digestion.

For external ulceration I have used the persesquinitrate of iron internally in a few cases, and apparently with benefit. Mr. Tindal writes me, that he gave it in a case of ulcer of the leg of eleven years duration, and effected a cure as he believes owing to its use. If these observations are correct, they do not contradict the experience respecting the aggravation of diarrhœa where ulceration of the mucous membrane of the intestine exists; because in one case the persesquinitrate does not touch the ulcer, in the other it does.

I have made some trials in scarlatina, with a view to prevent or mitigate ulceration of the fauces, but hitherto without any decided result.

In hiccough it is often a very potent remedy, and great benefit has been derived from it in tic doloureux.

Dr. Neligan mentions that Dr. Montgomery has used it very extensively in mucous discharges from the vagina, and in such cases considers it the best of the ferruginous preparations.

My friend Dr. Paton, of Paisly, who has had great experience with this medicine for many years, tells me that he has found it very useful in profuse mercurial salivation, both as a lotion and taken internally.

Chronic diarrhœa, however is the disease in which the persesquinitrate of iron has been longest and most extensively used. All my medical friends who have prescribed it, concur in saying, that it is very efficacious, and that many cases which had resisted all other treatment have been cured by it.

In making the solution of the persesquinitrate of iron, I now employ the following formula, which differs in a few respects from the original in the Edinburgh Medical and Surgical Journal.

Take of Iron Wire, (that sold under the name of No. 17) one ounce.
Nitric acid, three ounces by measure.
Water, fifty-seven ounces.
Muriatic acid, one drachm.

Mix the nitrc acid with fifteen ounces of water, (in very warm weather the quantity of water may be somewhat greater, and in cold weather somewhat less) in an earthen vessel capable of holding three or four times this quantity. Put into this diluted acid the iron wire broken into a number of pieces, and so twisted as to extend into every portion of the liquid. Cover the vessel lightly and set it aside. In eight or twelve hours the process is completed, when the solution is to be poured off the undissolved wire, and the remainder of the water together with the muriatic acid, added, to make up the whole sixty ounces, (thirty in the original formula.)

In this process there must be a slight excess of wire (say thirty grains) to ensure the combination of the whole of the acid. A great excess, if allowed to remain long in the liquid, would convert it into protonitrate. When properly prepared, the solution of the persesquinitrate of iron has a dark red color, like that of dark brandy; and the carbonate of soda produces a red precipitate, unmixed with any tinge of green. The taste is very astringent. The large quantity of water, and the free muriatic acid, are for the purpose of keeping the solution long transparent. In cold weather two or three months will elapse before it becomes muddy.—*Monthly Jour. of Medical Science.*

